# “Do as we say, not as we do?” the lifestyle behaviours of hospital doctors working in Ireland: a national cross-sectional study

**DOI:** 10.1186/s12889-019-6451-8

**Published:** 2019-02-11

**Authors:** Anthony O’ Keeffe, Blánaid Hayes, Lucia Prihodova

**Affiliations:** 1 Occupational Health Department, Beaumont Hospital, Dublin, Ireland; 2 Research Department, Royal College of Physicians of Ireland, Dublin, Ireland

**Keywords:** Lifestyle behaviours, Doctors, Hospital, Ireland

## Abstract

**Background:**

This study was conducted to assess the lifestyle behaviours of a national sample of hospital doctors working in Ireland. We also sought to compare the prevalence of these behaviours in doctors to the general Irish population.

**Methods:**

This was a national cross-sectional study of a randomised sample of hospital doctors working in Irish publicly funded hospitals and residential institutions. The final cohort consisted of 1749 doctors (response rate of 55%). All hospital specialties were represented except radiology. The following data were collected: sociodemographic data (age, sex), work grade (consultant, trainee) average hours worked over a two-week period, specialty and lifestyle behaviours (smoking, alcohol, physical activity). Lifestyle data for the general population was provided by the Healthy Ireland 2015 study.

**Results:**

Half of participants were men (50.5%). Just over half of the sample were consultants (54.3%), with 45.7% being trainees. 9.3% of doctors surveyed were smokers, 88.4% consumed alcohol and 24.5% were physically inactive. Trainees were more likely to smoke and be physically inactive when compared to consultants. Smoking rates amongst doctors were lower than the general population (9.3% -v- 23%). Doctors were more likely to consume alcohol than the general population (88.4% -v- 71.7%) but less likely to engage in binge drinking on a typical drinking occasion (12.8% -v- 39.5%). Doctors were more compliant than the general population with minimum exercise targets (75.5% -v- 70.5%), but less likely to engage in health enhancing physical activity (19.1% -v- 33%).

**Conclusions:**

While the prevalence of health behaviours amongst hospital doctors in Ireland compares favourably to the general population, their alcohol consumption and engagement in health enhancing physical activity suggest room for improvement. Continued health promotion and education on the importance of personal health behaviours is essential.

**Electronic supplementary material:**

The online version of this article (10.1186/s12889-019-6451-8) contains supplementary material, which is available to authorized users.

## Background

The deleterious health effects of poor lifestyle behaviours such as smoking, low physical activity and excessive alcohol intake are well established. Smoking has been shown to cause cardiovascular disease, pulmonary disease and cancer [1, 2] while excessive alcohol consumption has significant negative consequences for the health of individuals (e.g. cirrhosis, cancer, injuries) and society at large (e.g. drink driving) [3]. Physical inactivity is the third leading cause of death in the United States and contributes to the second leading cause (obesity), accounting for at least one in ten deaths [4].

Because of the impact of these behaviours, there has been an increase in research on doctors’ personal lifestyle behaviours in recent years. Evidence suggests that doctors’ wellbeing impacts on their ability to deliver effective care to their patients [5], and their personal lifestyle behaviours affect their preventative counselling of patients [6]. Doctors who engage in regular physical activity are more likely to counsel their patients regarding the health benefits of exercise [7]. Such counselling has been shown to be effective in improving the exercise habits of patients [8]. A consistent, positive relation between physicians’ and their patients’ preventive health practices (i.e. engaging in health screening programmes and vaccination uptake) has been demonstrated [9].

The health behaviours of doctors have also been shown to affect societal perception of the risk associated with these behaviours [10].

Although health care professionals have been shown to have lower rates of smoking and sedentary activity compared to other professions [11], there is considerable variation in the lifestyle behaviours of doctors internationally. The prevalence of smoking ranges from 2% in the US to 49% in Greece [12]. Only 29% of primary care doctors in Singapore achieve the minimum weekly exercise target of 150 mins/week [13], as opposed to 56.6% of primary care doctors in Northern Ireland [14].

Rates of alcohol misuse are comparable between doctors and the general population [15], and in some countries doctors engage in hazardous drinking at higher levels than the population at large [10]. In Bahrain, 98% of primary care doctors report lifelong abstinence from alcohol [16], whereas 91% of German hospital doctors consume alcohol [17]. These disparities are reflective of cultural differences in attitudes regarding the risks, benefits and appropriateness of the individual lifestyle behaviours and therefore are difficult to generalise.

To date, there has been little research on this topic performed in the Irish context [18]. Therefore, the aim of this study was to assess the lifestyle behaviours of a wide cohort of hospital doctors working in Ireland and to compare the prevalence of health risk behaviours in this group to that of the general working age population and a cohort that was matched for age, social class and level of education.

## Methods

### Design

This study was a national cross-sectional prevalence survey of hospital doctors in Ireland [19].

### Sample

A total sample of 3164 doctors, drawn from the registers of nine national post-graduate medical training bodies, was invited to participate in the study. The specialties represented were: anaesthetics, emergency medicine, medicine, obstetrics/gynaecology, ophthalmology, paediatrics, pathology, psychiatry and surgery. The Faculty of Radiology declined to participate. The sample included both consultants and trainees who were actively registered and working within a public hospital, public clinic or residential institution. Retirees, those practising exclusively in private practice and those working outside the jurisdiction of the Republic of Ireland were excluded from the study. In order to determine the sample size for inclusion in the postal questionnaire, the sample size for each subgroup (consultants and trainees within each specialty) within the total population was calculated for a 95% confidence interval, an acceptable margin of error of +/− 5% and an expected prevalence of 20% based on a review of the literature. This number was then doubled to allow for an estimated response rate of 50% rather than 100%.

The required sample size for each training body was calculated using Raosoft [20]. When this number exceeded the actual population sample size of the training body, randomisation was not applied. Ultimately, randomisation was only necessary for four of the 18 groups: medicine (trainees and consultants), psychiatric consultants and surgical trainees. Randomisation was applied in these four groups due to the large numbers of the groups they were drawn from and to ensure that the sample used in the study adequately represented the broader cohort. Randomisation was performed using the Microsoft Excel Randomisation Function.

### Procedure and methods

A self-administered questionnaire was used to assess smoking, alcohol consumption and physical activity (Additional file 1). The items in the questionnaire were largely modelled on the questionnaire used in the national survey of health and lifestyle behaviours of the Irish population – SLÁN/ Healthy Ireland survey [21]. The questionnaire was distributed by post and e-mail to all invited participants in April 2014. Two reminders were sent over the following 2 months, by post and e-mail.

An extensive participant information leaflet was included with the questionnaire outlining the purpose and scope of the study (Additional file 2). The leaflet also explained that the information was being gathered anonymously to ensure confidentiality and that the response to the questionnaire was indicative of consent.

### Demographic and work related information

The participants provided information on their sex, age, nationality, marital status as well as their work grade (trainee or consultant) and average hours worked per week over 2 weeks (under 40 h/week, 40–80 h/week, over 80 h/week).

### Smoking

Smoking habits were assessed using two questions. Respondents were asked whether they smoke and if so, how frequently (occasionally, daily).

### Alcohol

Alcohol consumption was assessed using three questions: frequency, number of standard drinks consumed on a typical drinking occasion and frequency of consuming six or more standard drinks on one occasion. Hazardous drinking behaviour was defined by consuming six or more standard drinks (60g of ethanol) on a single occasion, also described as “binge drinking” or “risky single occasion drinking” [22]. Hazardous drinking, not dependence, causes the majority of alcohol-related morbidity and mortality. Binge drinking accounts for almost one third of all motor vehicle deaths in the United States, as well as increasing patients’ risk for homicide, suicide, assaults, and nonintentional poisoning [23].

### Physical activity

The International Physical Activity Questionnaire Short Form (IPAQ-SF) was used to assess level of physical activity [24] using three categories: 1. inactive; 2 minimally active (completing at least 150 min of moderate intensity exercise per week over 5 or more days); and 3. health enhancing physical activity (HEPA; engaging in vigorous-intensity activity on at least 3 days achieving a minimum of at least 1500 MET-minutes/week (amount of energy expended carrying out physical activity)).

### Comparison with general population

To allow for comparison of health behaviours with the general population we used the Healthy Ireland 2015 survey dataset (which has superseded SLÁN). This annual national survey of the health and wellbeing of the general population includes individuals age 15 and over and gathers data on lifestyle behaviours, sexual health, general health and mental health [25]. In 2015, 7539 people were interviewed in the survey. To ensure comparability of the data, two groups were selected from the total cohort for comparison with our sample: 1. working-age general population of 25–65 years of age (*N* = 5209), and 2. population matched for age (> 25, < 65) and social class (Class 1 and 2) and level of education (3rd level) (*N* = 670).

There was a methodological difference between the two studies. The Healthy Ireland survey was interviewer administered, whereas ours was a self-administered questionnaire.

### Statistical analyses

Categorical variables were described by frequencies and percentages and between group differences were assessed with chi square tests. One-way analysis of variance (ANOVA) and independent t-tests were used to assess differences in means between groups. Binary logistic regression was used to assess whether respondents’ demographic and workplace variables were significant predictors for reporting lifestyle behaviours (smoking, binge drinking on a typical drinking occasion, physical inactivity). All dependent variables were dichotomised (presence or absence of consumption or activity). The explanatory variables included in each model were: sex, age group, work grade, nationality, marital status, mean hours worked over a 2 week period and specialty. IBM SPSS version 21 was used for the analysis of the individual cohorts (doctors and Healthy Ireland). Graphpad, an online statistical software programme, was used to test for differences between the doctor cohort and the comparator Healthy Ireland groups.

Participants who did not provide responses to specific questions were excluded from the subsequent analysis of the variable of interest.

The study protocol was approved by the Royal College of Physicians of Ireland’s (RCPI) Research Ethics Committee in December 2013 (RCPI RECSAF 20).

## Results

### Response rate

One thousand seven hundred forty nine doctors were included in the final analysis (55% response rate). Half of the participants were male (50.5%), the majority held Irish nationality (86%). Just over half of the sample were consultants (54.3%), with 45.7% being trainees. The majority of respondents were married or cohabitating (71.1%) (Table 1).

**Table 1:** Population characteristics and group differences in mean hours worked as tested by one-way ANOVA and independent t-test

Characteristic	% (N)	Mean hours worked/week (SD)
Age		
≤ 30	20.1 (349)	61.6 (13.1) ^***^
31–40	30.4 (529)	57.7 (15)
41–50	27 (469)	54.7 (15.7)
> 50	22.5 (391)	54.6 (15.1)
Sex		
Male	50.5 (882)	58.7 (15.4) ^***^
Female	49.5 (864)	55.3 (16.6)
Nationality		
Irish	86 (1505)	57.1 (15.1)
Non-Irish	14 (244)	56.6 (15.2)
Work Grade		
Consultant (C)	54.3 (950)	54.2^***^
Trainee (T)	45.7 (799)	60.4
Sex and Work Grade		
Male (C)	32.9 (574)	56.6 (14.9)^***^
Male (T)	17.6 (308)	62.6 (15.4)
Female (C)	21.5 (375)	50.4 (14.6)^***^
Female (T)	28 (489)	59 (13.5)
Marital status		
Married or cohabitating	71.1 (1224)	55.6 (15.1)^***^
Single	25.9 (445)	60.7 (13.6)
Divorced or separated	3 (52)	56.1 (18.4)
Specialty		
Anaesthetics	14.1 (247)	59.3 (14.4) ^***^
Emergency	4.9 (85)	53.6 (11.1)
Obstetrics & Gynaecology	6.2 (108)	59.7 (11.5)
Ophthalmology	2 (35)	55.1 (14.9)
Paediatrics	9.5 (165)	59.8 (14.9)
Pathology	8 (139)	49.9 (13)
Medicine	25.4 (433)	56.5 (12.7)
Psychiatry	16.1 (281)	47.6 (12.4)
Surgery	13.9 (243)	68.6 (17.1)

Between group differences: ^***^ *P* value ≤ .001

### Mean hours worked

The mean hours worked per week over a two-week period for the sample was 57 h/week (SD 15.1). Males worked on average 3.4 h more per week than their female counterparts. Consultants worked on average 6.2 h less per week compared to trainees. Doctors who were single worked 5 h more per week than their married/cohabitating colleagues. The highest and lowest mean weekly working hours by specialty were reported in surgery (68.6 h/week, SD 17.1) and psychiatry (47.6 h/week, standard deviation (SD) 12.4) respectively. The between group differences seen for age group, sex, work grade, marital status and specialty were statistically significant. There were no significant differences observed when compared by nationality (Table 1).

### Smoking

9.4% of the participants identified as smokers, with the majority being occasional smokers. Of the total cohort, 2.5% smoked daily. There was a significantly higher prevalence of smokers amongst the trainee group compared to consultants (*p* = 0.001) and amongst males compared to females (*p* = 0.007). Male trainees in particular demonstrated the highest rates of smoking (16.2%). Doctors who were single were significantly more likely to smoke compared to respondents who were married/cohabitating or divorced/separated (*p* = 0.000). The highest and lowest prevalence of smokers by specialty were observed in the emergency medicine doctors (18.8%) and paediatricians (6.1%) respectively (Table 2).

**Table 2:** Cross tabulation of demographic characteristics with smoking habits

Characteristic	Non-smoker % (N)	Occasional smoker % (N)	Daily smoker % (N)	X^2^
Age				
≤ 30	86.8 (302)	10.9 (38)	2.3 (8)	14.97^*^
31–40	90.9 (479)	7 (37)	2.1 (11)	
41–50	93.1 (432)	4.5 (21)	2.4 (11)	
> 50	90.7 (353)	5.9 (23)	3.3 (13)	
Sex				
Male	88 (778)	8.6 (75)	2.6 (23)	8.83^**^
Female	92.6 (796)	5 (43)	2.4 (21)	
Work Grade				
Consultant	92.7 (873)	4.8 (45)	2.5 (24)	13.77^***^
Trainee	88.2 (703)	9.3 (74)	2.5 (20)	
Sex and Work Grade				
Male (C)	91.5 (520)	6.2 (35)	2.3 (13)	12.93^**^
Male (T)	83.8 (258)	13 (40)	3.2 (10)	
Female (C)	94.4 (352)	2.7 (10)	2.9 (11)	8.01^*^
Female (T)	91.2 (444)	6.8 (33)	2.1 (10)	
Marital status				
Married or cohabitating	92.4 (1125)	5.1 (62)	2.5 (31)	21.57^***^
Single	86.2 (381)	11.1 (49)	2.7 (12)	
Divorced or separated	88.5 (46)	11.5 (6)	0 (0)	
Nationality				
Irish	90.9 (1360)	6.7 (100)	2.4 (36)	1.14
Non-Irish	88.9 (216)	7.8 (19)	3.3 (8)	
Specialty				
Anaesthetics	87.9 (217)	9.7 (24)	2.4 (6)	28.98^*^
Emergency	81.2 (69)	12.9 (11)	5.9 (5)	
Medicine	92.9 (407)	5.3 (23)	1.8 (8)	
Obstetrics & Gynaecology	86.9 (93)	8.4 (9)	4.7 (5)	
Ophthalmology	91.4 (32)	5.7 (2)	2.9 (1)	
Paediatrics	93.9 (155)	6.1 (10)	0 (0)	
Pathology	92 (127)	4.3 (6)	3.6 (5)	
Psychiatry	92.8 (259)	4.3 (12)	2.9 (8)	
Surgery	88.4 (214)	9.1 (22)	2.5 (6)	
Mean Hours Worked/Week				
< 40	88.5 (108)	6.6 (8)	4.9 (6)	6.31
40–80	91 (1287)	6.7 (95	2.2 (31)	
> 80	86.9 (113)	8.5 (11)	4.6 (6)	
Total	90.6 (1576)	6.8 (119)	2.5 (44)	

^*^P value <.05 ^**^P value <.01 ^***^P value ≤ .001

### Alcohol

11.4% of doctors surveyed were non-drinkers while 41.8% of the cohort reported consuming alcohol multiple times a week (Table 3). Of those who reported drinking alcohol, 27.5% never engage in binge drinking, while 10.1% did so at least once a week and a further 3.3% multiple times/week. Additionally, 12.8% reported binge drinking on a typical drinking occasion. The mean units of alcohol consumed on a typical occasion were 3.39 units (SD 2.46) (Table 3).

**Table 3:** Group differences in mean units of alcohol consumption as tested by one-way ANOVA and independent t-test and cross tabulation of demographic characteristics with patterns of alcohol consumption

Characteristic	Mean units of alcohol consumed on a typical drinking occasion		Binge drinking on a typical drinking occasion			Frequency of alcohol consumption					Frequency of binge drinking					
	MU	SD	Binges on a typical drinking occasion % (N)	Does not binge on a typical occasion %(N)	X^2^	Never % (N)	≤M % (N)	2–4/M % (N)	Multiple/W % (N)	X^2^	Never % (N)	<M % (N)	1–3/M %(N)	1/W %(N)	≥2/W %(N)	X^2^
Age																
≤ 30	4.9	3.3***	26.1 (71)	73.9 (201)	73.95***	10.3 (36)	16.9 (59)	46.7 (163)	25.8 (90)	171.5***	10.9 (34)	43.6 (136)	33.7 (105)	9.3 (29)	2.2 (7)	117.1***
31–40	3.5	2.6	15.2 (59)	84.8 (329)		17.8 (94)	18 (95)	34 (180)	30.1 (359)		25.6 (111	45.6 (198	19.4 (84)	6.2 (27)	3 (13)	
41–50	2.7	1.7	5.4 (20)	94.6 (347)		8.7 (41)	11.3 (53)	29.6 (139)	49.5 (232)		32.1 (136)	44.3 (188)	15.3 (65)	6.1 (26)	1.9 (8)	
> 50	2.7	1.4	6.6 (21)	93.4 (295)		6.9 (27)	9.7 (38)	21 (82)	62.1 (243)		38.6 (140)	35.5 (129)	13.2 (48)	6.1 (22)	6.3 (23)	
Sex																
Male	3.8	2.9***	19 (129)	81 (550)	46.75***	11.6 (102)	12.4 (109)	28.7 (253)	46.9 (414)	22.9***	21 (163)	40.7 (316)	22.7 (176)	9.5 (74)	5.9 (46)	81.3***
Female	2.9	1.9	6.6 (44)	93.4 (627)		11 (95)	15.9 (137)	36.2 (313)	36.6 (316)		34.1 (261)	43.7 (335)	17.1 (131)	4 (31)	0.7 (5)	
Work Grade																
Consultant	2.8	1.7***	6.6 (51)	93.4 (716)	60.02***	6.6 (63)	11.4 (108)	26.5 (252)	54.8 (521)	162.1***	33.9 (299)	41.2 (363)	14.3 (126)	6.4 (56)	3.9 (34)	66.3***
Trainee	4.2	3.0	20.9 (122)	79.1 (463)		16.9 (135)	17.4 (139)	39.4 (315)	26.2 (209)		19 (126)	43.6 (289)	27.3 (181)	7.4 (49)	2.6 (17)	
Sex and Work Grade																
Male(C)	3.09	1.91***	10.2	89.8	73.17***	7 (40)	11.5(66)	24(135)	57(329)		25(114)	43(198)	19 (88)	7.1(33)	6.1(28)	
Male (T)	5.45	3.77	37.8	62.2		20.1(62)	14 (43)	38(118)	28 (85)	86.2***	12.(27)	40(87)	32(70)	11(25)	3.7 (8)	27.3***
Female (C)	2.30	1.06***	1.3	98.7	24.92***	6.1 (23)	11.2 (42)	31(116)	51(124)	67.9***	48(147)	40(122)	8(25)	3 (9)	0 (0)	59.7***
Female (T)	3.45	2.18	10.9	89.1		14.7 (72	19.4 (95)	40(197)	25(316		24 (88)	47(173)	24(90)	3(13)	.8 (3)	
Marital Status																
Married or cohabiting	3.03	1.99***	8.9 (83)	91.1 (853)	50.79***	12.3 (150)	13.4 (164)	28.1 (344)	45.8 (560)	53.9***	30.3 (284)	44 (412)	16.8 (157)	5.6 (52)	3.2 (30)	37.9***
Single	4.44	3.24	23.9 (83)	76.1 (265)		9.4 (42)	15.3 (68)	44.9 (200)	30.3 (135)		18.7 (65)	42.2 (147)	29.3 (102)	7.2 (25)	2.3 (8)	
Divorced or separated	2.91	2.39	11.6 (5)	88.4 (38)		9.6 (5)	19.2 (1`0)	32.7 (17)	38.5 (20)		37.2 (16)	32.6 (14)	23.3 (10)	4.7 (2)	2.3 (1)	
Nationality																
Irish	3.5	2.5***	13.6 (166)	86.4(1054)	7.36***	7.3 (110)	13.4 (201)	34.2 (515)	44.7 (673)	191.6***	25.9 (360)	42.5 (590)	20.6 (286)	7.3 (101)	3.5 (49)	24.3***
Non-Irish	2.5	1.5	5.3 (7)	94.7 (125)		36.1 (88)	18.9 (46)	21.3 (52)	23.4 (57)		41.9 (65)	40 (60)	13.5 (21)	2.6 (4)	1.3 (2)	
Specialty																
Anaesthetics	3.6	2.9***	13.8 (27)	86.2 (168)	21.21**	9.3 (23)	15.4 (38)	31.6 (78)	43.7 (108)	47.2*	28.1 (63)	41.1 (92)	19.2 (43)	7.1 (16)	4.5 (10)	53.1 ns
Emergency	4.1	2.1	24.2 (16)	75.8 (50)		10.6 (9)	15.3 (13)	32.9 (28)	41.2 (35)		9.2 (7)	44.7 (34)	27.6 (21)	11.8 (9)	6.6 (5)	
Medicine	3.2	2.3	10.4 (34)	89.6 (293)		12.4 (55)	15.6 (69)	28.9 (128)	42.4 (188)		29.1 (112)	44.7 (172)	17.4 (67)	5.7 (22)	2.6 (10)	
Obstetrics & Gynaecology	3.5	2.3	13.8 (12)	86.2 (75)		8.3 (9)	9.3 (10)	38.9 (42)	42.6 (46)		25.5 (25)	42.9 (42)	23.5 (23)	5.1 (5)	3.1 (3)	
Ophthalmology	3.1	1.9	11.5 (3)	88.5 (23)		17.1 (6)	0 (0)	40 (14)	42.9 (14)		24.1 (7)	37.9 (11)	24.1 (7)	10.3 (3)	3.4 (1)	
Paediatrics	3.6	2.8	13.5 (17)	86.5 (109)		9.1 (15)	10.3 (17)	40.6 (67)	40 (6.6)		26 (39)	39.3 (59)	25.3 (38)	8 (12)	0.7 (1)	
Pathology	2.9	1.8	6.7 (8)	93.3 (112)		6.5 (9)	14.4 (20)	27.3 (38)	51.8 (72)		36.9 (48)	37.7 (49)	17.7 (23)	4.6 (6)	3.1 (4)	
Psychiatry	2.9	1.8	9.8 (21)	90.2 (193)		16.7 (47)	16 (45)	28.1 (79)	38.8 (109)		33 (77)	41.2 (96)	14.2 (33)	6.9 (16)	4.3 (10)	
Surgery	3.9	3	18.6 (35)	81.4 (153)		10.3 (25)	14 (34)	37.4 (91)	37.4 (91)		20.8 (45)	44.4 (96)	24.1 (52)	7.4 (16)	3.2 (7)	
Mean Hours Worked/Week																
under40	2.6	1.6***	92.9 (78)	7.1 (6)	5.31	13.8 (17)	16.3 (20)	22 (27)	46.3 (57)	18.8*	44.2 (46)	30.8 (32)	13.5 (14)	5.8 (6)	5.8 (6)	25.5**
40–80	3.4	2.5	87.3 (977)	12.7 (142)		11.1 (158)	13.4 (190)	32.8 (466)	42.4 (603)		26.8 (338)	42 (529)	20.4 (257)	7.1 (89)	3.3 (42)	
> 80	4.0	2.9	81.4 (79)	18.6 (18)		9.2 (12)	16.9 (22)	41.5 (54)	32.3 (42)		20.3 (24)	50 (59)	22 (26)	6.8 (8)	0.8 (1)	
Total	3.39	2.5	12.8 (173)	87.2 (1176)		11.3 (198)	14.1 (247)	32.4 (567)	41.7 (730)		27.5 (425)	42.2 (652)	19.9 (307)	6.8 (105)	3.3 (51)	

* *P* value <.05 ** *P* value <.01 *** *P* value ≤ .001 *MU* = Mean Units *M* = Monthly *W* = Weekly

There was a higher reported prevalence of non-drinkers amongst the trainee and non-Irish national groups, compared to their counterparts. The rates of abstinence were almost equivalent between males and females. Married/cohabitating doctors were more likely to abstain compared to their colleagues who were single or divorced/separated. Doctors working over 80 h/week had a higher rate of abstinence compared to those working under 40 h/week. By specialty, the highest and lowest prevalence of non-drinkers was reported amongst those practising ophthalmology and pathology respectively. The prevalence of drinking multiple times per week was significantly higher amongst consultants, males, those who were married/cohabitating, Irish nationals, those who worked under 40 h per week and doctors aged over 50, compared to their respective counterparts. The highest prevalence of drinking multiple times per week by specialty was reported by pathologists and the lowest was seen in surgeons (Table 3).

A significantly higher proportion of males engaged in binge drinking on a typical drinking occasion compared to their female counterparts (*p* = .000). 20.9% of trainees engaged in binge drinking on a typical occasion, compared with 6.6% of consultants (p = .000). Furthermore, 37.8% of male trainees engage in binge drinking on a typical drinking occasion, compared with 10.9% of female trainees (*p* = .0001). Only 1.3% of female consultants reported engaging in binge drinking on a typical occasion, significantly less than the 10.2% of male consultants (p = .0001). Single doctors were significantly more likely to binge drink on a typical drinking occasion compared to their married/cohabitating colleagues (p = .000). Binging on a typical occasion was significantly more prevalent amongst those holding Irish nationality. The highest and lowest prevalence of binging on a typical occasion were observed in doctors practising in emergency medicine and pathology respectively (Table 3).

### Physical activity

Almost one fifth (19.1%) of all doctors surveyed reported engaging in Health Enhancing Physical Activity (HEPA), while a quarter (24.5%) were inactive. Compared to females, males had a higher prevalence of HEPA and a lower prevalence of inactivity. Trainees had a higher prevalence of inactivity and a lower prevalence of HEPA than consultants. In particular, male consultants demonstrated a significantly lower prevalence of inactivity and a significantly higher prevalence of HEPA when compared to male trainees, female trainees and female consultants. When comparing based on specialty, the highest prevalence of HEPA was observed in anaesthetists, while ophthalmologists had the highest rates of physical inactivity. Doctors working more than 80 h per week engaged in HEPA at higher rates than their colleagues who worked less than 40 h per week. Rates of inactivity were almost equal in both groups (Table 4).

**Table 4:** Cross tabulation of demographic characteristics with levels of physical activity

Characteristic	Inactive	Minimally Active	HEPA	X^2^
Age				
≤ 30	22.1 (75)	56 (190)	21.8 (74)	34.13^***^
31–40	30.1 (153)	55.5 (282)	14.4 (73)	
41–50	25.6 (112)	58.1 (254)	16.2 (71)	
> 50	17.4 (64)	56.4 (207)	26.2 (96)	
Sex				
Male	21.2 (178)	57.9 (486)	20.9 (175)	11.22^**^
Female	27.9 (229)	54.9 (450)	17.2 (141)	
Work Grade				
Consultant	21.4 (190)	58.7 (521)	19.9 (177)	9.87^**^
Trainee	28 (217)	53.9 (417)	18.1 (140)	
Sex and Work Grade				
Male (C)	17.2 (93)	60.1 (325)	22.7 (123)	15.42^***^
Male (T)	28.5 (85)	54 (161)	17.4 (52)	
Female (C)	28 (97)	56.4 (195)	15.6 (54)	1.12
Female (T)	27.8 (132)	53.8 (255)	18.4 (87)	
Marital status				
Married or cohabitating	25.7 (298)	56.6 (656)	17.6 (204)	6.55
Single	22.4 (96)	55.4 (237)	22.2 (95)	
Divorced or separated	18.0 (9)	58 (29)	24 (12)	
Nationality				
Irish	23.5 (335)	57 (813)	19.6 (279)	5.95
Non-Irish	30.6 (72)	53.2 (125)	16.2 (38)	
Specialty				
Anaesthetics	15 (35)	58.1 (136)	26.9 (63)	37.40^**^
Emergency	27.5 (22)	55 (44)	17.5 (14)	
Medicine	23.7 (101)	54.9 (234)	21.4 (91)	
Obstetrics & Gynaecology	29.4 (30)	49 (50)	21.6 (22)	
Ophthalmology	32.3 (10)	48.4 (15)	19.4 (6)	
Paediatrics	22.2 (35)	57.6 (91)	20.3 (32)	
Pathology	25.2 (33)	62.6 (82)	12.2 (16)	
Psychiatry	30.9 (82)	56.2 (149)	12.8 (34)	
Surgery	25 (58)	58.6 (136)	16.4 (38)	
Mean Hours Worked/Week				
< 40	30.6 (34)	56.8 (63)	12.6 (14)	9.701^*^
40–80	23.1 (313)	57.3 (777)	19.7 (267)	
> 80	31.2 (39)	48 (60)	20.8 (26)	
Total	24.5 (407)	56.4 (938)	19.1 (317)	

^*^P value <.05 ^**^ *P* value <.01 ^***^ *P* value ≤ .001

### Logistic regression analysis

#### Smoking

The adjusted binary logistic regression model identified sex and marital status as significantly associated with smoking when controlling for specialty, age, work grade, mean hours worked per week and nationality (Table 5). Males were significantly more likely to smoke than females (OR 1.8; 95% CI -1.26-2.62). Doctors who were single were significantly more likely to smoke compared to their colleagues who were married/cohabitating (OR 1.76; 95% CI – 1.13-2.72) (Table 5).

**Table 5:** Adjusted odds ratio (95% CI) of variables associated with lifestyle risk factors

Characteristic	Smoking		Binge Drinking		Physical Inactivity	
	OR (CI)	*p*	OR (CI)	*p*	OR (CI)	*p*
Age						
≤ 30	.87 (.34–2.21)	.765	4.85 (1.8–13.04)	.002	.76 (.41–1.41)	.378
31–40	.60 (.26–1.35)	.214	2.06 (.88–4.79)	.095	1.20 (.72–2.01)	.48
41–50	.77 (.45–1.32)	.336	.93 (.47–1.84)	.839	1.49 (1.02–2.16)	.036
> 50	1		1		1	
Sex						
Male	1.8 (1.26–2.62)	.002	7.61 (4.82–12.03)	.000	.75 (.58–.97)	.026
Female	1		1		1	
Work Grade						
Consultant	.55 (.26–1.17)	.119	.57 (.26–1.23)	.151	.54 (.35–.86)	.008
Trainee	1		1		1	
Nationality						
Irish	1	.928	1	.001	1	.116
Non-Irish	1.02 (.62–1.68)		.25 (.11–.58)		1.31 (.94–1.83)	
Specialty						
Anaesthetics	1.07 (.59–1.96)	.822	.80 (.42–1.56)	.514	.57 (.34–.93)	.026
Emergency	1.94 (.94–4.01)	.075	1.24 (.55–2.78)	.604	1.22 (.66–2.26)	.522
Obstetrics and Gynaecology	1.45 (.70–3.02)	.317	1.03 (.45–2.35)	.946	1.21 (.68–2.14)	.516
Ophthalmology	.98 (.27–3.57)	.979	1.17 (.30–4.57)	.826	1.36 (.56–3.29)	.492
Paediatrics	.60 (.27–1.33)	.208	.87 (.40–1.86)	.709	.87 (.52–1.46)	.592
Pathology	.95 (.43–2.06)	.888	.68 (.28–1.66)	.398	1.08 (.63–1.86)	.772
Physicians	.69 (.38–1.30)	.214	.49 (.26–.93)	.03	1.04 (.68–1.57)	.868
Psychiatry	.68 (.34–1.37)	.284	.80 (.39–1.65)	.54	1.22 (.77–1.93)	.401
Surgery	1		1		1	
Mean Hours Worked						
< 40	1.58 (.67–3.71)	.295	1.28 (.40–4.17)	.677	.70 (.38–1.30)	.259
40–80	.86 (.46–1.59)	.63	1.35 (.66–2.76)	.41	.56 (.36–87)	.010
> 80	1		1		1	
Marital Status						
Married or cohabitating	1		1		1	
Single	1.76 (1.13–2.72)	.012	2.30 (1.44–3.67)	.000	.75 (.55–1.03)	.079
Divorced or separated	1.74 (.70–4.31)	.23	2.49 (.85–7.29)	.095	.52 (.28–1.21)	.128

#### Binge drinking on a typical drinking occasion

Sex, nationality, age group and marital status were significantly associated with binge drinking on a typical occasion in the binary logistic regression model when specialty, work grade and mean working hours per week were controlled for (Table 5). Males were nearly 8 times more likely to binge drink on a typical occasion compared with female colleagues (OR 7.61; 95% CI 4.82–12.03). Non-Irish nationals were four times less likely to binge drink compared to their Irish counterparts (OR 0.25; 95% CI 0.11–0.58). Doctors under 30 years of age were almost 5 times as likely to binge on a typical occasion compared to those over 50 (OR 4.85; 95% CI 2.34–15.89). Single doctors were more than twice as likely to binge drink on a typical drinking occasion when compared to their married/cohabitating colleagues (OR 2.3; 95% CI 1.44–3.67). Medical physicians were half as likely to engage in binge drinking on a typical drinking occasion compared to the reference group of surgeons (OR 0.49; 95% CI 0.26–0.93).

#### Physical inactivity

Sex, work grade, age group and mean hours worked were all significantly associated with physical inactivity in the adjusted binary logistic regression model when controlling for nationality, specialty and marital status (Table 5). Compared to females, males were significantly less likely to be inactive (OR 0.75; 95% CI 0.58–0.97). Consultants were significantly less likely to be inactive compared with trainees (OR 0.54; 95% CI 1.12–2.75). Doctors aged 40–50 were significantly more likely to be inactive compared to the reference group of doctors aged over 51 (OR 1.49; 95% CI 1.02–2.16). Doctors working 40–80 h/week were significantly less likely to be inactive compared to those working more than 80 h per week (OR 0.56; 95% CI 0.36–0.87). Anaesthetists were half as likely to be inactive as their surgical colleagues (OR 0.57; 95% CI 0.34–0.93); however, no other specialty was found to be significantly more or less likely to be inactive compared with the reference group of surgeons (Table 5).

### Comparison of lifestyle behaviours of doctors with general working age population and cohort matched for age, social class and education

The prevalence of daily smoking was significantly less in the doctor cohort compared to both the general working age population and the matched cohort (Table 6).

**Table 6:** Comparison of prevalence of lifestyle behaviours between hospital doctors working in Ireland and general working age population and cohort matched for age, social class and education

Characteristic	Doctors %	General working age population % (X^2^)	Matched Cohort % (X^2^)
Smoking			
Non-Smoker	90.6%	73.9% (211.01) ^***^	85.5% (12.56) ^***^
Occasional	6.8%	4.7% (12.06) ^***^	5.9% (.67)
Daily	2.5%	21.4% (332.13) ^***^	8.6% (43.27) ^***^
Alcohol			
Frequency of alcohol consumption			
Never	11.4%	18.3% (44.86) ^***^	9.6% (1.46)
Monthly or less	14.2%	25.1% (89.20) ^***^	19.2% (9.09) ^**^
2–4 times/month	32.5%	30% (3.91) ^*^	31.4% (0.27)
Multiple times/week	41.9%	26.6% (143.56) ^***^	39.8% (0.76)
Binges on a typical drinking occasion	12.8%	39.5% (326.97) ^***^	29.5% (78.33) ^***^
Mean units consumed on a typical drinking occasion	3.4	5.6 (SD: 4.16) ^***^	4.7 (SD: 3.47) ^***^
Physical activity			
Inactive	24.5%	29.5% (15.62) ^***^	26.3% (.847)
Minimally active	56.4%	37.5% (184.78) ^***^	39.9% (51.95) ^***^
Health enhancing physical activity	19.1%	33% (15.62) ^***^	33.8% (56.62) ^***^

Between group differences: ^*^P value <.05 ^**^ *P* value <.01 ^***^ *P* value ≤ .001 (Reference groups compared to doctors)

There were significantly more non-drinkers in the general population compared to the doctor cohort. The prevalence of drinking multiple times per week was significantly higher amongst doctors compared with the general working age population, but not compared to the matched cohort. Compared to doctors, the prevalence of binge drinking on a typical occasion was significantly higher amongst both the general working age population and the matched cohort. On average, doctors consumed significantly less alcohol on a typical drinking occasion than either the general working age population or the matched cohort.

Doctors engaged in HEPA at a significantly lower rate than both the general working age population and the matched cohort. However, their prevalence of physical inactivity was also significantly lower than in the general working age population.

## Discussion

This study aimed to assess the lifestyle behaviours of hospital doctors working in Ireland, and to compare the prevalence of these behaviours amongst doctors to that of the general working population in Ireland. The lifestyle behaviours of doctors have attracted significant attention in recent years. However, little is known about the Irish context and few studies have compared doctor’s lifestyle behaviours to those of the general population [10, 16]. The prevalence of smoking was less than 10%, which was in the mid-range for a developed nation – previous evidence shows that rates of smoking amongst hospital physicians range from 3% in Australia [12] to 15% in Denmark [26]. Consistent with much of the published data in this area, our results showed a significantly higher prevalence of smoking amongst males [12]. The findings from the adjusted model indicate that marital status is also significantly associated with smoking, with single doctors being significantly more likely to smoke compared with their married/cohabitating counterparts. This finding is consistent with previous studies of physicians [10] and also with studies of non-physician cohorts [27].

The majority of smokers smoked occasionally, and the prevalence of daily smoking was low at 2.5%. While daily smoking is associated with greater health risks, occasional smoking is associated with a significant increased risk of health problems when compared with non-smokers [28].

In line with previous studies showing lower smoking rates amongst health care professionals compared to other professionals [11], doctors were significantly less likely to smoke than either the general population or the matched cohort.

The rate of abstinence from alcohol was significantly higher amongst trainees compared with consultants, similar to trends observed in other European countries [29]. Consultants drank alcohol multiple times per week at a significantly higher rate than trainees but drank significantly lower volumes on a typical drinking occasion.

Irish nationals, males, doctors under 30 and doctors who were single were significantly more likely to binge on a typical occasion when compared to their respective counterparts. Our finding that male doctors tend to engage in harmful drinking at a significantly greater rate than females concurs with the majority of research in this area [10, 17], save for one recent study [30]. International evidence suggests that younger doctors tend to be more moderate in their drinking habits compared to their older colleagues [29], contrary to our findings. This discrepancy may reflect cultural differences amongst the general populations from which doctors are drawn as a similar pattern was observed in the Healthy Ireland study [25], which demonstrated that the prevalence of binge drinking in Ireland tends to decrease with age. Studies examining the relationship between the marital status of doctors and their patterns of alcohol use have demonstrated mixed results, with some finding that single doctors are more likely to engage in hazardous drinking when compared with their married colleagues [10, 15] and others finding the opposite relationship [30]. Surgeons were found to be twice as likely to engage in binge drinking as their medical colleagues. This concurs with previous research indicating higher rates of hazardous alcohol consumption amongst surgeons [17], however, this finding has not been consistently demonstrated [31].

When compared to the general working age population, there was a significantly lower prevalence of abstinence from alcohol amongst hospital doctors and they also reported higher prevalence of drinking multiple times per week. However, doctors reported drinking significantly less on a typical drinking occasion and were significantly less likely to binge on a typical drinking occasion compared to either group. Our findings suggest that while doctors in Ireland are more likely to be drinkers, they are less likely to engage in hazardous drinking behaviours. International data comparing doctors’ drinking habits to that of the general population has demonstrated mixed results, with results in Germany showing healthier habits amongst doctors [17], while results in Switzerland indicate more hazardous drinking amongst doctors [10].

The majority of doctors (75.5%) met the minimum weekly exercise recommendation of at least 150 min of moderate intensity exercise per week. Physical inactivity was significantly associated with sex, work grade, mean hours worked per week and one specialty. Males and consultants were significantly less likely to be inactive compared to their respective colleagues. Previous studies have also demonstrated a higher prevalence of inactivity amongst females [32] and trainees [33]. Those working more than 80 h per week were significantly more likely to be inactive compared to those working 40–80 h per week. Anaesthetists were significantly less likely to be inactive compared with their surgical colleagues, however, there were no significant difference in levels of inactivity between the remaining specialties and the reference category of surgeons. The prevalence of inactivity was lower in our cohort compared to previous international studies. For example, only 49.3% of Catalan doctors [34] and 53% of Brazilian doctors [35] were shown to meet the minimum weekly exercise target of 150 min/week.

Engaging in health enhancing physical activity was the main health behaviour identified in the study where the general population outperformed doctors. Compared with the general working age population and the matched cohort, doctors had a lower prevalence of physical inactivity; however, both comparison groups engaged in health enhancing physical activity at a significantly higher rate than doctors in Ireland. Studies comparing physicians’ exercise habits to those of their patients have demonstrated mixed results. Doctors in Bahrain and Catalonia are less physically active than their respective general populations [16, 34], whereas doctors in the U.S. engage in physical activity at higher rates than other professions in that country [36]. Previous studies have demonstrated an association between physical inactivity and long working hours [37]; however, this finding was not fully supported by our study. While doctors working over 80 h/week were more likely to be inactive compared to their colleagues working 40–80 h/week, they engaged in HEPA at higher rates than those working less than 40 h/week. Consequently, we cannot surmise with confidence that longer working hours explain the observed differences in rates of engaging in health enhancing physical activity between doctors working in hospitals in Ireland and the general population.

Some interventions aimed at improving the health behaviours of medical students and residents have shown positive results. “Self-awareness” and “self-care” interventions consisting of a lecture and written information about self-care habits, and a group discussion of self-care issues led to an improvement in the sleeping habits and activity levels of first year medical students compared to a control group who did not participate in the intervention programme. No change was observed in patterns of alcohol consumption [38]. An elective, team-based, 12-week, incentivized exercise programme designed for medical residents and fellows resulted in an increase in the percentage of people that met the recommendations for activity, a better quality of life and lower burnout scores compared to a control group that did not participate in the programme [39]. To our knowledge, no specific interventions targeted at medical students or residents have led to a reduction in the prevalence of binge drinking [23, 40, 41]; however, it has been suggested that using former impaired healthcare professionals to teach in medical schools and in hospitals may be an effective strategy [41]. Future research should investigate which strategies are most effective for decreasing alcohol misuse among physicians in training.

### Strengths and limitations

This study reports on findings from the first national survey of hospital doctors working within the same health system in Ireland and it is also the first study seeking to compare the lifestyle behaviours of doctors to those of the general population in Ireland. The response rate of 55% is reasonably high, given that studies involving doctors tend to have poor response rates [42]. The study included a wide cohort of hospital doctors representing almost all specialities found within the Irish health system and the sample size was deemed robust enough for the analysis performed; consequently, we are satisfied that our results are broadly representative of the target population; however, we do note that there was a higher representation of respondents holding Irish nationality when compared to the actual number of Irish graduates working within Irish hospitals [19], which may indicate some response bias.

The use of the same validated instruments for both our study and the Healthy Ireland study allow for ready comparison of many of the results.

However, due to methodological differences in how data relating to mean hours worked per week were recorded in the Healthy Ireland study and due to the poor response rate to this question in that study (< 10%), we could not accurately compare the mean working hours between our sample and the two reference groups.

Similarly, there was a slight methodological difference between the method used to assess alcohol consumption in this study and the one used in the Healthy Ireland survey. It has been demonstrated that people are more likely to underreport socially undesirable behaviours in the presence of an interviewer [43]. The sensitivity of the topic could have led to an underestimation of the prevalence of the relevant health risk behaviours in both our study sample and the general population data, as studies have demonstrated that sensitive questions provoke an increase in non-response and underreporting of socially undesirable behaviours [43]. To the best of our knowledge, no studies have addressed whether doctors are more or less likely to report socially undesirable behaviours than the general population. Similarly, we have not encountered any research into whether response rates to sensitive survey questions differs by medical specialty.

Aside from a longitudinal design, future studies should consider including other health behaviours, such as body mass index, dietary and sleeping habits and the use of illicit substances as well as their association with the likelihood of providing advice to patients on lifestyle behaviours. As this study was performed as part of a larger study exploring general and workplace wellbeing of doctors in Ireland [19], we were limited by the number of items used to assess health behaviours in order to avoid an excessively long questionnaire which might have impacted upon response rate.

## Conclusions

This study identified populations of hospital doctors in Ireland who were at increased risk of engaging in health risk behaviours. Male doctors were significantly more likely to smoke and engage in binge drinking compared to females. In particular, male trainees smoked and engaged in hazardous drinking behaviours at far greater rates than their colleagues. Trainees and female doctors were significantly more likely to be physically inactive when compared with their respective colleagues. Educational interventions addressing these issues targeted at these subgroups should be considered.

Overall, hospital doctors working in Ireland exhibit healthier lifestyle behaviours than both the general working age population and a cohort matched for age, socioeconomic status and level of education. This was demonstrated in lower rates of smoking, lower rates of hazardous alcohol consumption and lower levels of physical inactivity. However, their engagement in health enhancing physical activity was lower than the general population. This is an area in which doctors in Ireland could improve, as the increased health benefits associated with higher levels of physical activity are well established [44, 45].

The health and wellbeing of doctors is essential to the optimal functioning of a healthcare system [5]. Strong consideration should be given to implementing intervention programmes addressing the lifestyle behaviours of doctors, ideally during the pre-clinical years of undergraduate training.

## Additional files

Additional file 1: Study questionnaire; copy of the relevant sections of the questionnaire used in the study. (DOCX 133 kb)

Additional file 2: Information leaflet included with the questionnaire; this detailed the purpose and scope of the study. (DOCX 54 kb)

## Abbreviations

ANOVA: Analysis of Variance
C: Consultant
CI: Confidence Interval
HEPA: Health Enhancing Physical Activity
IPAQ-SF: The International Physical Activity Questionnaire Short Form
M: Monthly
MU: Mean Units
N: Number
OR: Odds Ratio
SD: Standard Deviation
T: Trainee
W: Weekly
