# Supplementary material for: “Do as we say, not as we do?” the lifestyle behaviours of hospital doctors working in Ireland: a national cross-sectional study
Source: BMC Public Health. 2019 Feb 11;19:179. doi: 10.1186/s12889-019-6451-8 (PMC6371571; doi:10.1186/s12889-019-6451-8)
Supplement: Supplementary file 1 — Study questionnaire; copy of the relevant sections of the questionnaire used in the study. (DOCX 133 kb) [file 12889_2019_6451_MOESM1_ESM.docx]

**RCPI Wellbeing Study Questionnaire**


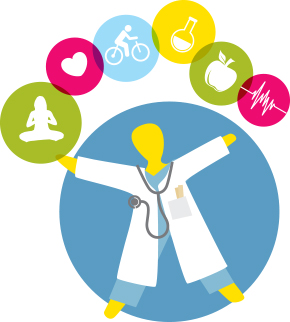


**Section 1: Demographic Details**

**A**

**1.0 How would you describe your current**

**employment?** HSE /Hospital Consultant without private work □

Hospital Consultant in private practice □

HSE Consultant with private work □

HST (Higher Specialist Training) □

BST (Basic Specialist Training) □

RTP (Registrar Training Programme) □

Other □ Please specify___________________________

- 1. **Which type of hospital are you attached to?**

Level 1 □ Level 2 □ Level 3 □ Level 4 □ Don’t know □ Not applicable □

**1.2** **If you are a consultant what was your year of appointment? ____________**

**1.3** **If you are a trainee what year did you start your current training programme?** ____________

**2.1** **What age were you when you completed your medical degree? ______ years**

**2. 2 What age are you now?** 25 or under □ 26 - 30 □ 31 - 40 □ 41 - 50 □ 51 – 60 □ 60+ □

**3. What is your gender?** Male □ Female □
**4. What is your marital status?** Married □ Single □ Separated □ Divorced □ Cohabiting □ Widowed □

**5. What is your nationality?** __________________

**6. What is your specialty? __________________**

**7. In which country was your medical school?** Ireland □ UK □ Europe □ Other/ Specify □________

**8. How would you describe your current work**

**commitment?** Full-time □ Part-time □ Specify _____________

**9. Do you do ‘on call’?**  Yes □ No □

**If yes, what is your standard rota?** 1:1□ 1:2□ 1:3□ 1:4 □ Other □ Specify________

**10. Over the past month, identify two consecutive**

**working weeks. How many hours per week**

**did you work in each?** Week 1 _________________ Week 2 _____________

**Section 3: Lifestyle**

**A**

**Please answer the following questions which relate to your alcohol intake:**

**1. How often do you have a drink containing alcohol?**

Never □ → Go to section **B** Monthly or less □ 2-4 times a month □ 2-3 times a week □ 4 or more times a week □

**2. How many standard drinks containing alcohol do you have on a typical day when you are drinking? ________________**

[A drink is: -A HALF PINT OR A GLASS OF BEER, LAGER OR CIDER

-A SINGLE MEASURE OF SPIRITS (E.G. WHISKEY, VODKA, GIN)

-A SINGLE GLASS OF WINE, SHERRY OR PORT

-BOTTLE OF ALCOPOPS (LONG NECK)]

**3. How often do you have six or more standard drinks on one occasion?**

Every day □ 5-6 times a week □ 2-4 times a week □ Once a week □ 1-3 times a month □ Less often □ Never □

**4. Over the past year have you driven a car after consuming 2 or more standard alcoholic drinks?**

Yes □ No □ Do not normally drive □

**B**

**Please answer the following questions that relate to smoking:**

**1.  Do you smoke tobacco products?**
Yes, daily □ →go to Q3       Yes, occasionally (less than daily) □→ go to Q3 Not at all □     →go to next section **C**                     
**2. Did you ever smoke?**
Yes, daily □         Yes, occasionally □  No, never smoked □    → go to next section **C**                

**3. Current Smokers only:  Number of Tobacco Products.**
On average, how many of the following do you smoke each day if you smoke daily or each week if you smoke occasionally?
a)    Manufactured cigarettes?         ____ per day OR ____ per week
b)    Hand-rolled cigarettes?            ____ per day OR ____ per week 
c)    Pipes full of tobacco?               ____ per day OR ____ per week

d)    Cigars?                                     ____ per day OR ____ per week 
e)    Any others?                             ____ per day OR ____ per week Specify: ____________________________

**4. If you are a current smoker (daily or occasional) are you?**
Trying to quit         □                                
Actively planning to quit           □                     
Thinking about quitting but not planning to                 □  
Not thinking about quitting                         □

**5. Do you use electronic cigarettes?**
 Yes, daily    □  Yes, occasionally (less than daily)   □      Not at all   □

**C**

**The following questions will ask you about the time you spent being physically active in the last 7 days. Please answer each question even if you do not consider yourself to be an active person. Please think about the activities you do at work, as part of your housework and in the garden, to get from place to place, and in your spare time for recreation, exercise or sport.**

| *Vigorous physical activities = hard physical effort which makes you breathe much harder than normal. Think only about these physical activities that you did for at least 10 minutes at a time.* |
| --- |

|  | |  |
| --- | --- | --- |
| 1. **During the last 7 days, on how many days did you do vigorous physical activities like heave lifting, digging, aerobics or fast cycling?**   ______ days None □   1. **How much time did you usually spend doing vigorous physical activities on one of those days?**   ________/ _______ hours and minutes per day Not sure / don’t know □ |  | |
|  |  | |
| *Moderate activities = moderate physical effort which makes you breathe somewhat harder than normal. Think only about those*  *physical activities that you did for at least 10 minutes at a time.* |  |  |
|  | |  |
| 1. **During the last 7 days, on how many days did you do moderate physical activities like carrying light loads, bicycling at a regular pace, or doubles tennis? [Do not include walking].**   _______ days None □ |  | |
| 1. **How much time did you usually spend doing moderate physical activities on one of those days?**   ________/ _______ hours and minutes per day Not sure / don’t know □ |  | |
| *Think about the time spent walking in the last 7 days. This includes at work and at home, walking to travel from place to place, and any other walking that you might do solely for recreation, sport, exercise or leisure.* |  |  |
|  | |  |
| 1. **During the last 7 days, on how many days did you walk for at least 10 minutes at a time?**   ______ days None □ |  | |
| 1. **How much time did you usually spend walking on one of these days?**   ________/ _______ hours and minutes per day Not sure / don’t know □ |  | |

**Section 6: Feedback**

**We encourage you to use the space below to add any comments you may have about any aspect of doctors’ wellbeing, stress, measures to prevent stress, access to supports and any other issues you believe are relevant to this study. If the space is insufficient, please feel free to attach additional page(s).**

**We plan to undertake further qualitative research in autumn 2014 by means of focus groups and interviews. These will explore your perspectives on maintaining wellbeing and coping in your current work environment. If you think you may be interested in participating in this further wave of research, please email your name and contact details to** [**wellbeing@rcpi.ie**](mailto:wellbeing@rcpi.ie) **to register your interest.**

**Please place the completed questionnaire in the brown stamped addressed envelope to post at your earliest convenience.**


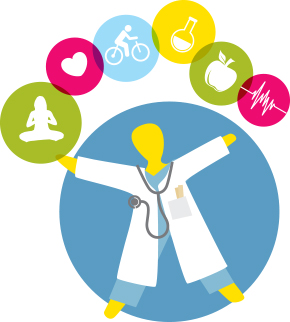

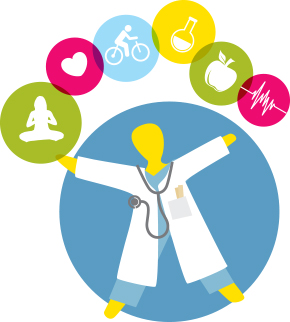


**Thank you for taking the time to complete this questionnaire.**
