# Supplementary material for: “Do as we say, not as we do?” the lifestyle behaviours of hospital doctors working in Ireland: a national cross-sectional study
Source: BMC Public Health. 2019 Feb 11;19:179. doi: 10.1186/s12889-019-6451-8 (PMC6371571; doi:10.1186/s12889-019-6451-8)
Supplement: Supplementary file 2 — Information leaflet included with the questionnaire; this detailed the purpose and scope of the study. (DOCX 54 kb) [file 12889_2019_6451_MOESM2_ESM.docx]

**Appendix 7: Postal Questionnaire Information Leaflet**


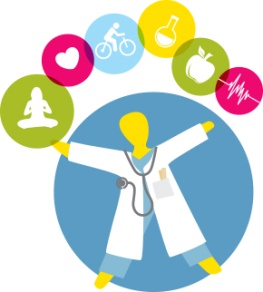


| **Study title: *A national study of wellbeing of hospital doctors in Ireland*** |
| --- |

**Principal investigator’s name: Dr. Blánaid Hayes**

**Email address of principal investigator:** [blanaidmhayes@physicians.ie](mailto:blanaidmhayes@physicians.ie)

**Co-investigator’s name: Ms. Gillian Walsh**

***Email address of co-investigator:*** [WalshG@RCPI.IE](mailto:WalshG@RCPI.IE)

You are invited to take part in a research study to be carried out at the Royal College of Physicians of Ireland (RCPI). This is a national, randomised, cross-sectional questionnaire study.

Before you decide whether or not you wish to take part, you should read the information provided below carefully and, if you wish, discuss it with a member of your family, a friend or a colleague. Take time to think about it and don’t feel under pressure to make a quick decision.

You should clearly understand the rationale for this study and any risks and benefits of taking part so that you can make a decision that you are happy with. By responding to the questionnaire either electronically or by post, you are consenting to participate.

You don't have to take part in this study and there will be no penalty if you don’t.

| **Why is this study being done?** |
| --- |

This research study is being carried out to assess the wellbeing of Irish hospital doctors with a view to informing future interventions for maintaining health and accessing preferred care. There has been little research into the health and wellbeing of doctors in this country. There are many reasons why one might expect that doctors in Ireland experience stress in their working lives. The changing structures of the national ‘health service’ as well as new organisational structures (e.g. the directorate model) have had an impact on how people work. The financial and personnel constraints imposed by recession translate into greater work volume, tighter deadlines and dissatisfaction of service users. All of this is stressful for those working to provide patient care. More specifically, the Irish Government and HSE’s failure to implement the European Working Time Directive (EWTD) to date is undoubtedly contributing to malaise and low morale, particularly in non-consultant hospital doctors (NCHDs).

Some of the questions which we hope this research will answer include:

1. What is the level of psychological distress in a population of Irish hospital doctors?
2. Is there a difference between doctors who report high levels of distress and those who don’t (e.g. age, gender, specialty and geographical location)?
3. Are lifestyle health risks of hospital doctors in Ireland comparable to those in the general population (controlled for SE status)?
4. Are measures of psychological distress related to work stress?
5. What are the key workplace stressors for hospital doctors in Ireland?
6. What are the most common coping strategies used by hospital doctors in Ireland?

| **Who is organising this study?** |
| --- |

This research is being conducted by two researchers (named above) under the auspices of a multi-stakeholder Steering Group based in the RCPI. The Steering Group is comprised of representatives from the Royal College of Physicians of Ireland, the Royal College of Surgeons in Ireland, the College of Anaesthetists of Ireland, the College of Psychiatrists of Ireland, the Faculty of Radiology and the Irish Association of Emergency Medicine. Each professional body has influenced the design of the study protocol and questionnaire from the outset.

| **Why am I being asked to take part?** |
| --- |

You are being asked to take part in this study because you are registered as either a consultant or trainee with one of the national medical training bodies. Only doctors who work in Irish hospitals are being included in this study. You will receive the questionnaire if you have been selected to participate through a process of randomisation. Your training body has been involved in the study’s design from an early stage and has facilitated access for the researchers to the database for the purpose of this randomised selection.

| **How will the study be carried out?** |
| --- |

Approximately 4,000 people (consultants and trainees) will receive a copy of the questionnaire (both by post and by email) in April 2014. Information on the study (including this leaflet) is now accessible on the RCPI website at [www.rcpi.ie/wellbeing](http://www.rcpi.ie/wellbeing) *.* All completed questionnaires should be returned in the stamped, addressed envelopes provided for analysis or electronically if preferred. A low response rate may introduce bias and doubt about how generally the findings may be applied.

| **What will happen to me if I agree to take part?** |
| --- |

If you agree to take part you will be asked to set aside 15-25mins in order to complete the questionnaire which you will submit either electronically or by post. Both options will be available to you. Please choose just one.

The questionnaire includes a number of standard instruments which will measure the prevalence of stress, depression, anxiety and burnout as well as work stress, wellbeing, workability, lifestyle behaviours and some questions on stigma and career satisfaction.

By using a coding card, we will ensure that respondents to the first wave will not receive subsequent postal reminders to participate. In addition, the card will enable us to ensure that respondents receive their reward (see below). All respondents will receive an electronic copy of the study results.

This is an important Irish study which will be of interest to doctors themselves and to all others who share an interest in improving the working lives of hospital doctors and enhancing patient care. We expect analysis to be completed by the end of 2014.

| **What are the benefits?** |
| --- |

Many doctors engage in research on patients / other subjects but have little opportunity to be the subject of research themselves. The key benefit of your contribution to this project is that the findings will be published with a view to highlighting the key workplace stressors experienced by hospital doctors in Ireland and their experience of wellbeing. A report on the results of this research will be presented to all the participating professional bodies and the Forum of Post Graduate Training Bodies with a view to determining how it can be used to improve working conditions for Irish hospital doctors. The results will be used to inform future developments regarding supports and pathways to care for doctors.

| **What are the risks?** |
| --- |

The demand on time is the only risk envisaged for most of those choosing to take part in this study. Therefore, we suggest that if you wish to respond, you put aside the designated time within a week of receipt of the questionnaire so that it doesn’t get overlooked.

A small number of respondents may experience unease and / or upset upon reading the questionnaire, since by its very nature, the issues explored are sensitive. The HSE has funded a single point of contact counselling service for the duration of this study which may be accessed at [ray.maloney1@hse.ie](mailto:ray.maloney1@hse.ie) . The researchers have also prepared a ‘Wellbeing Pack’ which we direct you to should you feel anxious or distressed by any of the questions. This pack is readily accessible on [www.rcpi.ie/wellbeing](http://www.rcpi.ie/wellbeing) .

| **Will it cost me anything to take part or will I be rewarded in any way?** |
| --- |

You will not incur any costs by participating in this study. If you choose to return the questionnaire by post, the stamp is already paid for.

We are providing a pen in trainee packs and all trainee respondents will be entered into a draw for an iPad. We propose a non-monetary ‘reward’ to consultant respondents in the form of 1 X Internal CPD credit under Practice Evaluation. This has been approved by RCPI’s Professional Competence Department. In addition all consultants will be entered into a draw for a €300 restaurant voucher.

| **Is the study confidential?** |
| --- |

As this study seeks answers to very sensitive questions responses will be 100% anonymous. The following measures will be put in place to ensure confidentiality of information:

- No names or personally identifiable information will be collected. Demographic information about age, gender, speciality, nationality and year of training (where applicable) will be sought. However, no information will be collected which could link respondents to their individual questionnaire.
- A key card will be used for the sole purpose of tracking non-responders and for dissemination of ‘rewards’ (see above). Codes will be assigned to individual respondents prior to the mailing of surveys and a corresponding key card will be returned with the un-coded questionnaire. Upon return, key cards will be separated from the questionnaire by an administrator external to the research team. Under no circumstances will codes be accessed on an individual basis. Nor will they be available to the researchers. The key linking these ID codes to the identity information of participants will be kept secure and separate from the returned data in a password-protected file in the Research Department of RCPI. This will be accessible only to an assigned independent external administrator. This individual will provide the research team with the codes of those who have not responded to the survey after a specified time period following each postal wave of the survey. This file will be permanently deleted once all 3 waves have been completed.
- At no stage of the study shall either the researchers or the independent administrator be able to identify the questionnaires of individual respondents. Therefore the researchers will be unable to return surveys to individual respondents even if this is requested.
- Data from the study will be published in aggregate form. No individual can or will be identified. No identifying information will be used in any subsequent reports, presentations or publications.
- Individual electronic and paper data collected will be destroyed 7 years after completion of the analysis and only the collective data will be held.
- All data will be collected and maintained in accordance with legislative requirements (Data Protection Act 1988 and Amendment 2003).

| **What will happen to the results?** |
| --- |

- A detailed knowledge exchange and dissemination (KED) plan has been outlined in the study protocol. All respondents will receive an electronic copy of a summary of the study findings. A research report will also be sent to participants and placed on the research website of all participating training bodies. All other consultants and trainees will be alerted by email to this report. Any peer-reviewed publication arising from the study will also be placed on the research websites of the participating professional bodies.
- The report will be printed and circulated to all training bodies and other interested parties (e.g. Department of Health, Irish Medical Council, HSE) to be agreed in consultation with the Forum. Offers of presentation of findings at study days / specialty conferences will be made to all participating training bodies (e.g. St. Luke’s Day Symposium RCPI).

**If you have any further questions about this study please contact either:**

[blanaidmhayes@physicians.ie](mailto:blanaidmhayes@physicians.ie) or [anncoughlan@rcpi.ie](mailto:anncoughlan@rcpi.ie)

Thank you for taking the time to read this information leaflet. We do hope that you will agree to participate.
